# Distribution and abundance of hematophagous flies (Glossinidae, *Stomoxys*, and Tabanidae) in two national parks of Gabon

**DOI:** 10.1051/parasite/2015023

**Published:** 2015-07-17

**Authors:** Paul Yannick Bitome Essono, François-Xavier Dechaume-Moncharmont, Jacques Mavoungou, Régis Obiang Mba, Gérard Duvallet, François Bretagnolle

**Affiliations:** 1 Université de Bourgogne, UMR 6282-Biogéosciences 6 Boulevard Gabriel 21000 Dijon France; 2 Institut de Recherche en Écologie Tropicale (IRET-CENAREST) BP 13354 Libreville Gabon; 3 Centre de Recherche Médicale de Lambaréné, Albert Schweitzer BP 118 Lambaréné Gabon; 4 UMR 5175 CEFE, Université Paul-Valéry Montpellier, Route de Mende 34199 Montpellier Cedex 5 France

**Keywords:** Hematophagous flies, Distribution, Climatic seasons, National Parks, Gabon

## Abstract

In order to minimize risks of pathogen transmission with the development of ecotourism in Gabon, a seasonal inventory has been performed in five contrasted biotopes in Ivindo (INP) and Moukalaba-Doudou (MDNP) National Parks. A total of 10,033 hematophagous flies were captured. The Glossinidae, with six different species identified, was the most abundant group and constitutes about 60% of the captured flies compared to the *Stomoxys* (6 species also identified) and Tabanidae with 28% and 12%, respectively. The Glossinidae showed a higher rate of capture in primary forest and in research camps. In INP, the *Stomoxys* showed a higher rate of capture in secondary forest and at village borders, whereas in MDNP the *Stomoxys* were captured more in the savannah area. Thus, each fly group seemed to reach maximum abundance in different habitats. The Glossinidae were more abundant in primary forest and near research camps while *Stomoxys* were more abundant in secondary forest and savannah. The Tabanidae did not show a clear habitat preference.

## Abstract (fr)

Afin de minimiser les risques de transmission de pathogènes avec le développement de l’écotourisme au Gabon, un inventaire saisonnier a été mené dans cinq biotopes caractéristiques des parcs nationaux de l’Ivindo (PNI) et de Moukalaba-Doudou (PNMD). Au total, 10 033 mouches hématophages ont été capturées. Les Glossinidae, avec 6 espèces différentes identifiées, constituaient le groupe le plus abondant avec 60 % de mouches capturées, suivi des *Stomoxys* (6 espèces différentes identifiées) et des Tabanidae avec respectivement 28 % et 12 %. Les glossines ont été majoritairement capturées en forêt primaire et au niveau des camps de recherche. Au PNI, les stomoxes ont été majoritairement capturés en forêt secondaire et en périphérie des villages. Alors qu’au PNMD, ces stomoxes ont été majoritairement capturés au niveau des savanes. Ainsi, chaque groupe de mouches semble être inféodé à un type de milieu. Les glossines préfèrent la forêt primaire et camps de recherche, alors que les stomoxes préfèrent la forêt secondaire et la savane. Les tabanides ne dégagent aucune préférence particulière pour un type de milieu.

## 1. Introduction

Hematophagous flies, and among those, species belonging to the families Glossinidae and Tabanidae and to the *Stomoxys* genus, play an important role in human health and wild and domestic animal health because many species of these groups are vectors of organisms responsible for several human and animal diseases [5, 49, 54]. Moreover, several vector-transmitted diseases are considered as emergent due to their recent evolution and their propagation. The preponderant role of the species belonging to the Glossinidae family in the transmission of African Human Trypanosomiasis (AHT), or sleeping sickness, has historically hidden the potential role of other hematophagous flies, like those belonging to the genus *Stomoxys* and the family Tabanidae, in trypanosome transmission [12, 13, 32] and the transmission of other pathogens. For example, it is now recognized that several species of the genus *Stomoxys* are vectors of parasites, such as *Trypanosoma* sp., and various viruses, such as the *Capripox-viruses* responsible for lumpy skin disease in sheep and goats [6, 8]. The Tabanidae are also mechanical or biological vectors of many human and animal pathogens [4, 11] and an analogous pattern of trypanosome transmission has been documented for several tabanids of the *Atylotus* genus [1, 11]. Species of the *Chrysops* genus are involved in the cyclical transmission of *Loa loa* filariasis [50].

Since the 1980s, an exponential increase of emergent infectious diseases (EID) has been observed in the world [55, 56], among which the recent increase or the re-emergence of parasitic infections in humans and domestic animals. It is now widely accepted that recent progression of ancient zoonoses is connected to ecological factors which result from environmental changes generated by human activities [10, 30]. In particular, in the tropical regions, the increase of EIDs is associated with the dramatic alteration of natural ecosystems (deforestation, poaching, and bushmeat consumption) and to the increasing human encroachment into wild areas that were previously free of human settlements [3, 27, 48]. The recent development of ecotourism can also promote this process of disease emergence. Currently, new empirical approaches that emphasize the ecology of potential vectors of EIDs are being developed to understand and to quantify the contamination risk in various situations [41]. In particular, the implementation of area-wide integrated pest management principles has recently been successfully tested on *Glossina palpalis gambiensis* in Senegal [15]. The implementation of such methods needs a complex modelling approach fuelled by a full understanding of the ecology of the vector species.

Hematophagous fly activity is highly seasonal [4, 9]. For example, most of the *Glossina* species respond to seasonal patterns, and within a region, the populations of the different species increase in the rainy season [22]. In Uganda, Harley [26] found that most of the Tabanidae and *Stomoxys* species were caught throughout the year but that there was a seasonal rise in abundance corresponding to the ends of the rains and the beginning of the dry season. However, the basic seasonal pattern of the different groups is influenced by local climate parameters and species exhibit various patterns of population fluctuation related to local climate, vegetation, and host blood meal source [9, 37]. Hence, the design of management strategies against the different species and of strategies that minimize the risk of fly bites requires a full regional understanding of the species’ phenology and ecology.

These last 10 years, the government of Gabon has developed an ambitious conservation program for its natural areas based on the development of national parks (which constitute about 11% of the national territory) and the development of ecotourism as one of its economic strategies. However, the human presence in previously human-free areas linked to the presence of several diseases (due to bacteria, viruses, protozoa, etc.) could enhance the risk of transmission of emergent diseases, especially where hematophagous flies have dense populations.

The chorology and the ecology of hematophagous flies in Central Africa and in Gabon have been partially studied. Several publications have addressed the chorological status of the tsetse species in Central Africa [16, 39, 40] and the ecology of tsetse species has been extensively studied in many parts of Africa [9]. These studies showed that the majority of the tsetse species of Central Africa are dependent on forest cover although some species can be found at low frequency in open habitats depending on the hygrometry [9, 57]. Moreover, a strong seasonal pattern of abundance is found, the abundance of the flies increasing at the end of the rainy season and reaching its maximum at the beginning of the dry season [9]. The stomoxes and the tabanids showed higher densities in secondary forest, open habitats, and in the vicinity of anthropized areas [5, 43, 44]. However, the knowledge of the environmental parameters that govern the distribution and abundance of hematophagous flies needs to be increased in Gabon in order to design wide area pest management strategies for local and tourist populations and to minimize interactions between humans and hematophagous flies. The present study was conducted to improve our understanding of the distribution, the abundance, and the phenology of hematophagous flies (Glossinidae, *Stomoxys*, and Tabanidae) in two national parks of Gabon.

## 2. Materials and methods

### 2.1. Study zone

**Figure 1.**
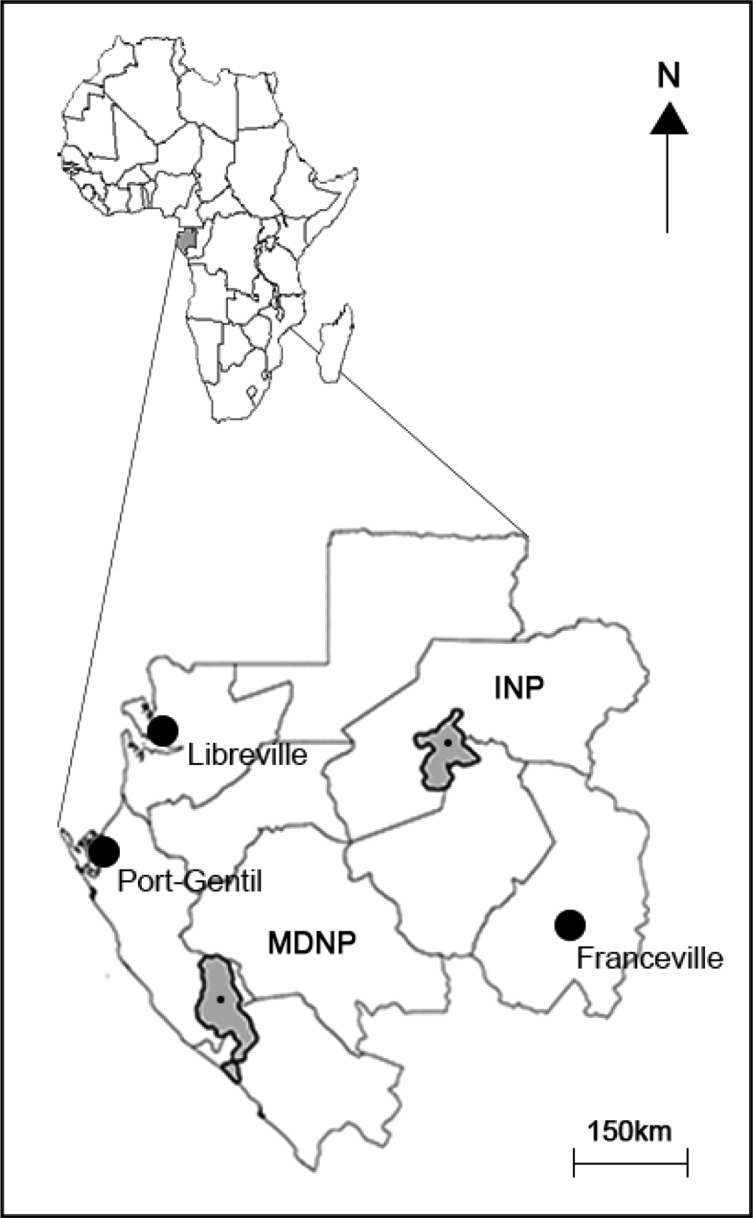
Illustration of the study zone: Ivindo and Moukalaba-Doudou national park.

The study areas consisted of two Gabonese national parks 450 km apart from one another (Fig. 1); the Ivindo National Park (INP) (N: 00°30.828′/E: 12°48.201′) and the Moukalaba-Doudou National Park (MDNP) (S: 00°24.475′/E: 10°34.203′). The INP, located in the north-east of the country, is largely dominated by primary forest with minor patches of open wetlands and secondary forests. Some villages are established at the border of the park and a research camp (IPASSA Research Station) is set up within the park. The MDNP, located in the south-western part of the country, is dominated by a mosaic of savannah and primary forest. A research camp (BOTSIANA Research Station) is established within the park and the park is bordered by a few villages.

The climate of Gabon, humid and equatorial, is characterized by two alternating dry and rainy seasons. The short dry season (SDS) occurs from mid-December to mid-March and is followed by the long rainy season (LRS) from mid-March to mid-June. The long dry season (LDS) occurs from mid-June to mid-September and ends with the small rainy season (SRS) from mid-September to mid-December.

### 2.2. Sampling strategy

Hematophagous flies were sampled over almost 2 years between 2012 and 2014 in order to cover the four climatic seasons (SDS, LRS, LDS, SRS) in the two national parks. Each sampling campaign consisted of a series of seven consecutive days of capture during each climatic season and in each park. Flies were collected in five contrasted habitats: (1) the primary forest (PF) is a forest with a closed canopy and hence with a shrub layer at low density; (2) the secondary forest (SF) is a forest with an open canopy and with a very rich and dense shrub layer dominated by the family Marantaceae; (3) the research camps (RCs) are places where the forest has been cut down over a small area in order to establish a scientific research station (IPASSA in INP and BOTSIANA in MDNP); (4) the savannahs (SAVs) are wide herbaceous vegetation sites, and (5) the villages (VGs) around the parks are anthropized areas. However, the savannah habitat was not present in the INP and the secondary forest was not sampled in the MDNP.

Hematophagous flies were captured with two different traps: the Vavoua and the Nzi traps. The Vavoua trap was initially developed for the capture of Glossinidae and was also used for the capture of *Stomoxys* on Réunion Island [19, 36]. The Nzi trap was more adapted to the capture of *Glossina pallidipes* and tabanids in Africa [2, 46]. At each sampling point, the two traps were installed side by side (1 Vavoua and 1 Nzi) at a distance of 1 m. Although the proximity of the two traps could influence the catches, this sampling strategy was decided in order to maximize the catch of the different species. Three sampling points were spaced out with intervals of 500 m following a transect in the five biotopes described above. Each season, we used 24 traps (12 Vavoua and 12 Nzi) over 7 days in each national park: 6 traps in research camps, 6 traps in primary forests, 6 traps in villages, 6 traps in secondary forests (only in INP), and 6 traps in the savannah (only in MDNP). The capture effort was calculated as the number of traps installed by day in the parks and over the four seasons: (24 traps × 7 days × 4 climatic seasons × 2 national parks) = 1344 traps-days.

### 2.3. Data analysis

We calculated the apparent density by trap (ADT) index in order to estimate the relative abundance of each species in the different sampled biotopes and in each season. The ADT index was calculated as: ADT = number of captured flies × (number of traps × number of days of capture)^−1^. To assess the influence of biotopes and season on the number of flies captured we used generalized linear models with mixed-effect and model selection procedures based on information theory [18, 35]. As a first step, we analyzed the absolute number of flies considered at the level of the taxonomic group (Glossinidae, *Stomoxys*, and Tabanidae). Then, we analyzed each group separately in order to assess differences among species within each group. We used a zero inflated generalized mixed linear model with a negative binomial distribution using the statistical package “glmmADMB” [17] for R software version 3.0.2 [52]. In each park, an *a priori* full model was defined including the taxonomic group (Glossinidae, *Stomoxys*, and Tabanidae), the season, the biotope, and the two-way interactions between these co-variates. We considered the taxonomic group, the season, and the biotope as fixed effects and the trapping sites as a random effect. This full model was compared to the simpler models taking into account some of the co-variates or their interactions [18, 35]. The best models were ranked according to the Akaike information criterion (AIC_c_), the Akaike weights (*w*) and differences (Δ) between the minimum AIC_c_ and AIC_c_ for a given model were used to compare competing models [18, 35]. The Akaike weight of a given model varies between 0 and 1 and models with the largest weight (and lowest delta) are the most plausible models. As a second step, separated analysis within each taxonomic groups (Glossinidae, *Stomoxys*, and Tabanidae) was carried out following the same procedure as described above in order to analyze differences of abundance among species. We considered an *a priori* full model taking into account the species, the season, and the biotopes (with their two-way interactions) as co-variates and the trapping sites as random effect.

## 3. Results

**Figure 2.**
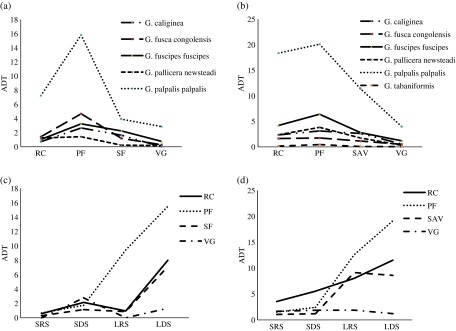
Distribution (ADT) of tsetse flies: Tsetse species according to four biotopes in (a) INP (RC: research camps, PF: primary forest, SF: secondary forest, VG: villages) and (b) MDNP (RC: research camps, PF: primary forest, SAV: savannah, VG: villages); Glossinidae family caught during the four climatic seasons (SRS: small rainy season, SDS: short dry season, LRS: long rainy season, LDS: long dry season) in (c) four biotopes (RC: research camps, PF: primary forest, SF: secondary forest, VG: villages) of INP and (d) four biotopes (RC: research camps, PF: primary forest, SAV: savannah, VG: villages) of MDNP.

**Figure 3.**
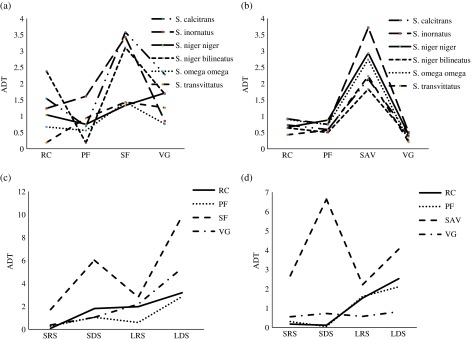
Distribution (ADT) of stomoxes: *Stomoxys* species according to four biotopes in (a) INP (RC: research camps, PF: primary forest, SF: secondary forest, VG: villages) and (b) MDNP (RC: research camps, PF: primary forest, SAV: savannah, VG: villages); *Stomoxys* genus caught during the four climatic seasons (SRS: small rainy season, SDS: short dry season, LRS: long rainy season, LDS: long dry season) in (c) four biotopes (RC: research camps, PF: primary forest, SF: secondary forest, VG: villages) of INP and (d) four biotopes (RC: research camps, PF: primary forest, SAV: savannah, VG: villages) of MDNP.

**Figure 4.**
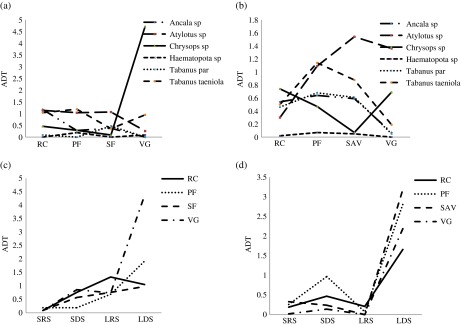
Distribution (ADT) of tabanids: Tabanidae species according to four biotopes in (a) INP (RC: research camps, PF: primary forest, SF: secondary forest, VG: villages) and (b) MDNP (RC: research camps, PF: primary forest, SAV: savannah, VG: villages); Tabanidae family caught during the four climatic seasons (SRS: small rainy season, SDS: short dry season, LRS: long rainy season, LDS: long dry season) in (c) four biotopes (RC: research camps, PF: primary forest, SF: secondary forest, VG: villages) of INP and (d) four biotopes (RC: research camps, PF: primary forest, SAV: savannah, VG: villages) of MDNP.

A total of 10,033 hematophagous flies were captured in 1344 traps-days. We captured 4554 flies (45.39%) in Ivindo National Park “INP” (Table 1) and 5479 flies (54.61%) in Moukalaba-Doudou National Park “MDNP” (Table 1). The Glossinidae family was the most abundant and constituted about 60% of the captured flies, compared to the *Stomoxys* and Tabanidae with 28% and 12%, respectively. Considering the variation of the different taxonomic groups along seasons and biotopes, in both trapping sites, our model selection procedure indicated that the best model (i.e. the model with the lowest AIC) was obtained with the full model (i.e. main effects and the interactions of the groups with biotopes or seasons). In MDNP, the Akaike weight was *w* = 0.992 whereas the second best model (the full model without biotopes × seasons interaction) reaches a weight of *w* = 0.08 (with a Δ = 9.59), which means that only the full model can be considered as the best model: we observed an effect of every co-variate and their two-way interactions. In MDNP, the Akaike weight of the full model was *w* = 0.674, whereas the second best model (the full model without groups × seasons interaction) reaches a weight of *w* = 0.29 (with a Δ = 1.67). These results indicate that the different families of hematophagous flies showed heterogeneous variation of abundance along seasons and biotopes. In both national parks, the Glossinidae showed higher rates of capture in primary forest (ADT = 7.0 [±*SD* 18.2] in INP; ADT = 9.0 [±*SD* 19.32] in MDNP) and in research camps (ADT = 3.0 [±*SD* 7.11] in INP; ADT = 7.2 [±*SD* 14.87] in MDNP) (Figs. 2a and 2b). In INP, the *Stomoxys* showed the highest rates of capture in secondary forest (ADT = 5.12 [±*SD* 6.61]) and in villages (ADT = 2.24 [±*SD* 1.97]) whereas in MDNP the *Stomoxys* were more frequently captured in savannah (ADT = 5.0 [±*SD* 1.91]). The Tabanidae did not show a clear habitat preference (Figs. 4a and 4b). We captured 4433 flies (45%) in long dry season (LDS), 3187 flies (31%) in long rainy season (LRS), 1317 flies (13%) in short dry season (SDS), and 1096 flies (11%) in small rainy season (SRS). In each park and in each biotope, the model selection procedure described above revealed a strong and significant variation in the abundance of the total amount of flies captured during the different seasons. In both national parks, the tsetse and the tabanids showed a consistent increase of abundance throughout the year (Figs. 2c, 2d, 4c and 4d), being far less abundant during small rainy (SRS) and short dry season (SDS) and then increasing in long rainy season (LRS) and in long dry season (LDS). For the stomoxes (Figs. 3c and 3d), a different pattern was observed. Two peaks of abundance were observed (SDS and LDS), suggesting that this group has an optimum in dry periods. Thus, each group seemed to reach maximal abundance in different habitats and seasons. The Glossinidae were more abundant in a closed vegetation habitat during the long rainy and the following long dry seasons, whereas *Stomoxys* were more abundant in more open habitats and during the dry seasons.

**Table 1.** The apparent density (ADT) of hematophagous flies distributed in three groups: Glossinidae, *Stomoxys* and Tabanidae in Ivindo (INP) and Moukalaba-Doudou (MDNP) national parks for the four climatic seasons sampled (SRS: small rainy season, SDS: short dry season, LRS: long rainy season, LDS: long dry season) and for five biotopes (RC: research camps, PF: primary forest, SF: secondary forest (in INP), SAV: savannah (in MDNP), VG: villages). *N* represents the number of flies species caught by group. The second column (%) gives the proportion of each species/genus belonging to that group of flies.

INP	%	ADT															
		SRS				SDS				LRS				LDS			
		RC	PF	SF	VG	RC	PF	SF	VG	RC	PF	SF	VG	RC	PF	SF	VG
**Glossinidae (*N* = 2206)**		**0.58**	**0.67**	**0.29**	**0**	**2.14**	**1.74**	**1.14**	**2.77**	**0.95**	**9.43**	**0.86**	**0**	**8**	**15.5**	**7.12**	**1.33**
*G. caliginea*	8.7	0.09	0	0.09	0	0.14	0.09	0.09	0	0.09	0.95	0.19	0	0.4	1.62	1.26	0
*G. fusca congolensis*	14.42	0	0	0	0	0.57	0.38	0.09	0.19	0.09	0.66	0.38	0	0.76	3.62	0.72	0.09
*G. fuscipes fuscipes*	14.23	0	0.09	0	0	0.09	0	0.19	0.48	0	0.29	0.19	0	0.95	2.86	2.05	0.28
*G. pallicera newsteadi*	5.89	0	0	0	0	0	0.19	0.09	0.09	0	0.19	0.05	0	1.24	1.04	0.09	0.09
*G. palpalis palpalis*	56.75	0.48	0.57	0.19	0	1.33	1.07	0.66	2	0.77	7.81	0.05	0	4.66	6.36	3	0.85
***Stomoxys* (*N* = 1732)**		**0.09**	**0.28**	**1.72**	**0.38**	**1.81**	**1.05**	**6.02**	**1.05**	**1.98**	**0.6**	**2.81**	**2.19**	**3.19**	**2.86**	**9.86**	**5.33**
*S. calcitrans*	21.25	0	0	0	0	0.09	0.19	0.66	0.09	0.35	0.12	0.48	0.28	1.1	0.38	3.1	1.91
*S. inornatus*	23.33	0.09	0.19	1.33	0.19	0.38	0.19	1.14	0.19	0.48	0.09	1.33	0.19	0.29	1.14	2.1	0.28
*S. niger niger*	15.24	0	0	0.19	0.19	0.57	0.09	1.33	0.09	0.09	0.09	0.38	1.24	0.38	0.57	0.76	0.19
*S. niger bilineatus*	18.01	0	0	0	0	0.48	0.09	1.07	0.09	0.66	0.09	0.14	0.09	1.24	0	1.9	1.5
*S. omega omega*	12.7	0	0.09	0.19	0	0.29	0.38	1.62	0.28	0.19	0.09	0.19	0.19	0.19	0	1.05	0.48
*S. transvittatus*	9.47	0	0	0	0	0	0.09	0.19	0.09	0.19	0.09	0.29	0.19	0	0.76	0.95	0.98
**Tabanidae (*N* = 616)**		**0.09**	**0.19**	**0.09**	**0.09**	**0.76**	**0.19**	**0.57**	**0.86**	**1.33**	**0.69**	**0.76**	**0.74**	**1.05**	**1.92**	**0.98**	**4.36**
*Ancala* sp.	13.96	0.09	0	0	0	0.19	0.09	0	0	0.19	0	0	0	0	0.19	0.38	0
*Atylotus* sp.	27.27	0	0	0	0	0.09	0.09	0	0	0.19	0	0.57	0.19	0.86	0.95	0.5	0.07
*Chrysops* sp.	29.22	0	0	0	0.09	0.28	0	0	0.48	0.09	0.29	0.09	0.45	0.09	0	0	3.7
*Haematopota* sp.	0.32	0	0	0	0	0	0	0	0	0	0.19	0	0.09	0	0	0	0
*Tabanus par*	2.27	0	0	0	0	0.09	0	0.47	0	0	0	0	0	0	0	0	0
*Tabanus taeniola*	26.95	0	0.19	0.09	0	0.09	0	0.09	0.38	0.86	0.21	0.09	0	0.09	0.79	0.09	0.57
MDNP	%	ADT															
		SRS				SDS				LRS				LDS			
		RC	PF	SAV	VG	RC	PF	SAV	VG	RC	PF	SAV	VG	RC	PF	SAV	VG
**Glossinidae (*N* = 3833)**		**3.55**	**1.52**	**1.05**	**1.66**	**5.54**	**2.42**	**1.19**	**1.86**	**8.02**	**12.57**	**9.14**	**1.88**	**11.52**	**19.12**	**8.6**	**1.21**
*G. caliginea*	9.81	0.14	0.16	0.19	0.24	0.64	0.55	0.19	0.12	0.83	0.91	0.88	0.07	0.74	1.5	1.36	0.14
*G. fusca congolensis*	5.77	0.36	0.29	0.19	0.24	0.69	0.26	0.12	0.16	0.26	0.31	0.21	0.07	0.33	0.88	0.62	0.02
*G. fuscipes fuscipes*	16.59	0.07	0.12	0	0	0.45	0.4	0.03	0.26	1.73	3.16	1	0.33	1.95	2.71	1.74	0.6
*G. pallicera newsteadi*	8.01	0.21	0.07	0.07	0.16	0.36	0.28	0.04	0	0.83	1.88	0.88	0.02	0.97	1.59	0.74	0.14
*G. palpalis palpalis*	58.86	2.76	0.88	0.6	1.02	3.4	0.92	0.52	1.3	4.36	6.3	6.17	1.38	7.83	11.98	4.07	0.26
*G. tabaniformis*	0.97	0	0	0	0	0	0	0	0	0	0	0	0	0.16	0.45	0.07	0.04
***Stomoxys* (*N* = 1105)**		**0.17**	**0.3**	**2.67**	**0.55**	**0.11**	**0.04**	**6.64**	**0.72**	**1.52**	**1.6**	**2.21**	**0.57**	**2.52**	**2.1**	**4.05**	**0.81**
*S. calcitrans*	14.93	0	0	0.31	0.07	0	0.02	0.93	0.12	0.26	0.21	0.4	0	0.47	0.36	0.5	0.26
*S. inornatus*	22.99	0.07	0.12	0.79	0.09	0.02	0.02	1.93	0.09	0.4	0.35	0.4	0.12	0.43	0.26	0.6	0.21
*S. niger niger*	18.82	0.07	0.02	0.5	0.02	0	0	1.3	0.07	0.26	0.36	0.5	0.26	0.33	0.5	0.64	0.09
*S. niger bilineatus*	12.85	0.02	0.09	0.31	0.07	0.07	0	0.52	0.07	0.12	0.17	0.12	0.12	0.43	0.24	0.88	0.12
*S. omega omega*	17.19	0	0	0.5	0.02	0.02	0	1.12	0.07	0.33	0.24	0.33	0.04	0.55	0.5	0.79	0.12
*S. transvittatus*	13.21	0	0.07	0.26	0.02	0	0	0.83	0.17	0.14	0.26	0.45	0.02	0.29	0.24	0.69	0
**Tabanidae (*N* = 541)**		**0.19**	**0.26**	**0.33**	**0.02**	**0.47**	**0.97**	**0.24**	**0.14**	**0.21**	**0.07**	**0**	**0**	**1.66**	**2.81**	**3.19**	**2.19**
*Ancala* sp.	14.42	0.12	0	0.02	0	0	0.12	0	0	0	0	0	0	0.42	0.52	0.57	0.07
*Atylotus* sp.	33.46	0.02	0.07	0.07	0	0.07	0.21	0.07	0	0.12	0	0	0	0.09	0.81	1.4	1.36
*Chrysops* sp.	15.34	0	0	0	0	0	0	0	0.14	0	0	0	0	0.74	0.47	0.07	0.55
*Haematopota* sp.	0.92	0	0	0	0	0.02	0.07	0.05	0	0	0	0	0	0	0	0	0
*Tabanus par*	14.79	0.02	0.07	0.07	0	0.21	0.26	0.09	0	0.09	0	0	0	0.14	0.35	0.45	0.05
*Tabanus taeniola*	21.07	0.07	0.12	0.167	0.02	0.17	0.31	0.02	0	0	0.07	0	0	0.26	0.64	0.69	0.17

A total of 14 species were identified, 6 species of Glossinidae, 6 species of *Stomoxys*, and 2 species of Tabanidae. The remaining Tabanidae were identified at the genus level. We captured six different tsetse species in MDNP (Table 1) whereas one species (*G. tabaniformis*) was never recorded in our traps in INP (Table 1). In both sites sampled, *G. palpalis palpalis* was the most abundant species and represented almost 60%. The second most abundant species, in both sites, was *G. fuscipes fuscipes* (respectively, 14 and 17% in INP and MDNP). *Glossina fusca congolensis* was captured at a higher frequency in INP (14.42%) than MDNP (6%) whereas the two remaining species (*G. caliginea* and *G. pallicera newsteadi*) showed comparable frequencies of capture between the two sites (<10%). Concerning the stomoxes, we captured six different species in both national parks. The relative abundance of the different species was homogeneous and no species clearly dominated the community of trapped insects. Finally, three genera or species were dominant in tabanids, the genus *Atylotus*, the genus *Chrysops* and *Tabanus taeniola* again with a relative homogeneity of abundance among the two national parks.

Within the tsetse flies, the best model to explain the species abundance variation in MDNP was the full model without the species × biotopes interaction (Akaike weight *w* = 1, the second best model had a delta = 18) whereas in INP the best model was the full model (Akaike weight *w* = 0.994) and the second model, the full model without species × biotopes interaction was far less plausible (weight *w* = 0.06 with a Δ = 10.4). Hence, these results suggest that the relative abundance of the tsetse species significantly varied among biotopes and seasons. However, these interactions result mostly from the amplitude of abundance variation of *G. palpalis* and from the higher abundance of this species in savannah and villages, whereas the other species are rare in these biotopes (Fig. 2). Apart from this species, the other tsetse species showed a relatively homogeneous response to biotope and season, being more abundant in forested biotopes and increasing in densities in long rainy seasons (Fig. 2). Within the stomoxes, the only plausible model in MDNP was the additive model (taking into account only the three main effects without the interactions) with a weight *w* = 0.99 (the second best model Δ = 9.8), whereas in INP the only plausible model was the full model with all the interactions (weight *w* = 1, second best model Δ = 23.4). The relative abundance fluctuations among stomoxe species illustrated in Figure 3 show that in MDNP all the species varied homogeneously along biotopes and seasons, being far more abundant in savannah and in dry seasons. In INP, the relative abundance was less homogeneous according to biotope and season, although the dry seasons and the open natural habitats were the most favorable. Within the tabanids, the estimation of standard deviation of the parameters of the full model with all the interactions (species × season and species × biotope) was never possible due to the number of zero, the inflation of degree of freedom, and the sample size requested with such a model. Hence the best model in both parks was the additive model with species, biotopes, and seasons (MDNP weight *w* = 1, second best model Δ = 11; INP weight *w* = 1 and second best model Δ = 63). Our data suggest that a strong heterogeneity of abundance exists for the different genera or species according to biotopes and seasons (Fig. 4). However, a general pattern of increased abundance during the long rainy season similar to the tsetse was observed for the different taxonomic units, i.e. low abundance in SRS and SDS and progressive increase in LRS, especially in MDNP (Figs. 4c and 4d).

## 4. Discussion

In the present study, we analyzed the variation of abundance of different families of hematophagous flies in Gabon in order to complement and to increase the knowledge on the ecology of these families in Central Africa. Although the different families of flies were captured during our study, we found a heterogeneous distribution of abundance among biotopes and seasons for the different captured hematophagous fly species. The Glossinidae was the dominant family captured and represented 60% of the trapped individuals whereas the *Stomoxys* and the Tabanidae represent 28% and 12%, respectively. However, it is very hard to assess whether this apparent heterogeneity reflects the real relative abundance of the different taxonomic groups. Indeed, a possible bias could result from the differential attractiveness of the trap used toward the different groups in interaction with the environmental parameters (for example, light intensity) [2, 19, 36, 46]. Six species and subspecies of genus *Glossina* were identified in the present study. This finding corroborates data reported by other authors in the region [24, 39, 40, 58]. However, we did not identify *G. tachinoides*, a species reported in the INP by Zinga et al. [59] and in MDNP by Dibakou et al. [14]. The possible presence of this xeric species normally reported in Central Africa, in northern parts of Cameroon [25, 53], and of Central African Republic [16] needs to be more deeply documented. Moreover, Dibakou et al. [14] have reported the presence of *G. nashi* in Doussala village and research camp in Moukalaba-Doudou National Park, while this species is almost absent in any environment frequented by humans [29, 38].

The ecology of the tsetse flies has been widely studied in West, East, and Central Africa [9, 16, 34, 53]. These studies suggest an ecological specialization for many species, some being adapted to forest habitats, whereas other species are more dependent on riverine or savannah habitats. However, most of the *Glossina* species tend to avoid disturbed and open areas and show a discontinuous distribution determined by environmental criteria that include abiotic factors (density of the vegetation, rainfall, temperature, and saturation deficit) and host blood meal source [20, 33, 37]. In our study, the most captured species, *G. p. palpalis*, was found on almost all biotopes sampled, although in other parts of Africa, this species tends to occur in riverine and lacustrine habitats. This trend was also found by other authors in Central Africa where *G. p. palpalis* was the dominant species captured [21, 23]. All the tsetse species sampled in our study occur sympatrically, predominantly in forested biotopes and along the different seasons. In the literature, the tsetse guild, i.e. the cohabitation of several tsetse species that exploit the same resource, comprise generally two or three species [22]. In the present study, we found a very rare situation of five or six different coexisting species in the same biotopes. In Ivory Coast, a study found that the conflicting coexistence of several species results from a dynamic and complex equilibrium that has been partially investigated [22]. One possible explanation for the coexistences of so many species could result from a segregation of diurnal activity. For example, *G. p. palpalis* is mostly active between 11 h and 16 h [31], whereas *G. tabaniformis* is predominantly active at the beginning and the end of the day [53]. Such diurnal segregation could promote the coexistence of the species, although this has not been fully documented [9]. The complex tsetse guilds present in Gabon would provide an interesting model to deeply study the dynamics of coexistence.

The *Stomoxys* represented 28% and were more abundant in open biotopes as the secondary forest, savannah, or villages. Concerning these hematophagous flies, we found six different species in our study. Mavoungou et al. [43] captured seven different species that were identical to our sampling, although we did not capture *Stomoxys xanthomelas*. However, this species occurred at a very low frequency in their study (0.04% of about 16,000 stomoxes sampled) and is highly associated with the forest canopy [42, 45]. The ecological niche of this species and the necessity to capture the flies on the canopy probably explain why we were unable to sample this species in the present study. In our study, the relative abundance of the different *Stomoxys* species did not show a clear pattern. The most abundant species were *S. inornatus* (24%) and *S. calcitrans* (19%), whereas the least abundant species was *S. transvittatus* (10.8%). These results differ slightly from the abundance obtained by Mavoungou et al. [43] for the same set of species in Gabon where the most abundant species were *S. niger niger* (33%) and *S. transvittatus* (33%), the other species being far less abundant. These differences could be explained by various factors. The genus *Stomoxys* shows different ecological preferences. For example, *S. calcitrans* is a cosmopolitan species associated with human activities and is frequently found in anthropized environments [60]. In their study in Kenya, Mihok et al. [47] also found a low frequency of this species in forest and woodland. Moreover, we found that most of *Stomoxys* species were trapped in secondary forest or savannah. These results are consistent with what has been published concerning the ecology of these flies and where most species are predominantly found in secondary forest [43, 45, 47]. It is hence possible that differences between the biotopes sampled and the season, when sampling was carried out, are the main drivers of the incongruity between our results and those previously published. In Uganda, Harley [26] found that *Stomoxys omega* was more abundant in forested areas and *Stomoxys niger niger* and *S. calcitrans* did not show a clear preference. A sampling bias due to the light conditions in secondary forest could explain observed differences in published results, because higher luminosity in more open habitats could enhance the attractiveness of the traps.

The Tabanidae family represents 12%, but did not show a clear association with the different biotopes and climatic seasons sampled; it is difficult to extract a clear pattern. We identified six genera and two species, including *Tabanus par* and *Tabanus taeniola*. Different genera identified are known in the region [28] with a predominance of the genus *Tabanus* [44]. Some tabanid species are present in all the biotopes but predominate in only one biotope or season. This is the case for the genus *Chrysops* that predominates in the village during LDS in INP. The genus *Tabanus* is known to be abundant in many biotopes and throughout the different seasons [28, 44, 51]. In a study in the East of Gabon, Mavoungou et al. [44] found that the *Tabanus* species were mostly associated with forested biotopes, in particular secondary forest, whereas the *Chrysops* genus was mostly trapped in anthropized areas and villages. In Uganda, Harley [26] found that the Tabanidae of the genera *Tabanus* and *Ancala* were more abundant in open habitats. However, the multiple differences observed in our study concerning the species composition of hematophagous flies compared to previously published data probably result from the sensitivity of each species to environmental regional parameters. The low relative frequency of tabanids captured in our study could result either from the lower density of this taxonomic group in the biotopes sampled or from the low attractiveness of our traps. Some genus-specific trends are known to be associated with the various traps that have been designed for the capture of hematophagous flies including tabanids [4, 44]. The Vavoua trap is efficient for *Chrysops* sp. whereas the Nzi trap is more general for tabanids. However, these two traps have been designed for the capture of tsetse flies and stomoxes and may not be optimal to attract tabanids.

The seasonal pattern of fly abundance is a well-known phenomenon and has been described for all the families studied in the present study. For example, the different Glossinidae species show seasonal fluctuation roughly related to rainfall distribution with an increase of populations when rains start and a decrease along the dry season [9, 22]. However, this basic pattern is modulated by the local climatic parameters such as the length of the rainy season and the distribution of rains. As a result, the different Glossinidae species exhibit various patterns of population fluctuations related to local climate and vegetation [20, 23]. The fluctuations of abundance related to rain have also been described in the *Stomoxys* and Tabanidae species [5]. Tabanid activity is highly seasonal in the tropics. Frequently, a peak is observed during the rainy season although huge interspecific differences occur [4]. This probably explains why no clear pattern of seasonality could be extracted from our data. The seasonal pattern generally shows an increase of abundance during the rainy season although the different species can be captured throughout the year at lower abundance.

## 5. Conclusion

Up to recently, tsetse flies have been considered the most important taxonomic group among the African hematophagous flies due to their preponderant role in the transmission of African human trypanosomes. However, recently, the role of other hematophagous flies, like those belonging to the stomoxes and tabanids, in the transmission of many pathogens to humans and cattle has been reconsidered and these species are now recognized as the vector of many parasites, bacteria, and viruses [3, 9, 10]. In this context, enhancing the knowledge of the parameters that determine the density fluctuations of the various hematophagous flies in Central Africa is a fundamental prerequisite to minimize the contamination risk for human populations by the various pathogens for which these species have vectorial competence. The recent development of ecotourism in the different national parks of Gabon is also an emerging concern because it will increase the human presence in natural areas where hematophagous flies are present in high density. In the present study, we have provided new data on the variation of the different species’ distribution and density according to biotopes and seasons in two national parks of Gabon. Our results confirm that the various species probably have different ecological optima, as shown by their pattern of occurrence in the traps along the different seasons but also that the densities of insects reach a minimum during the dry seasons. This could help to design optimal strategies to minimize the fly challenge to humans in these natural areas.
